# Mast Cells in Human Health and Diseases

**DOI:** 10.3390/ijms24076668

**Published:** 2023-04-03

**Authors:** Giovanna Traina

**Affiliations:** Department of Pharmaceutical Sciences, University of Perugia, 06126 Perugia, Italy; giovanna.traina@unipg.it

This Special Issue includes articles that discuss several important aspects regarding the role of mast cells (MCs) and elucidate some cellular and molecular mechanisms of these multifaceted cells. Many studies have reported and demonstrated that MCs play crucial roles in normal immune responses and disease states.

Mitochondria have been reported to be involved in MC activation. In article [1], the authors demonstrated that mitocurcuminoids cause mitochondrial fragmentation, do not affect spontaneous cell degranulation and inhibit antigen-dependent degranulation. Mitochondrial fragmentation inducers can cause mitochondria to malfunction so that they are unable to perform their functions during antigen-dependent activation of MCs. These results help us to understand the mechanisms of action of mitochondrial-derived curcuminoids on MCs and their mitochondrial functions and suggest the development of new drugs for inflammatory and allergic diseases.

Obesity is associated with a high prevalence and occurrence of knee osteoarthritis (KOA), and synovial neuropeptides may contribute to this disease. Neuromedin U (NMU) is an example of a synovial neuropeptide and plays roles in multiple physiological functions such as inflammation, stress response, circadian rhythmicity and feeding behavior. Experiments studying genetic ablation or overexpression of NMUs have demonstrated the presence of crosstalk between the NMU system and obesity-related pathological factors. NMUR1 receptor mRNA is present in T cells, natural killer cells, eosinophils and MCs. Study [2], conducted on KOA patients, found that NMUR1 expression was elevated in the synovial membrane of obese patients with osteoarthritis compared to normal weight patients. NMUR1 expression was found in MC fractions, suggesting that MCs may contribute to the NMU/NMUR system in the osteoarthritic synovium of obese patients.

In article [3], an investigation was directed into the signaling induced by the alarmin interleukin-33 (IL-33), which is released following cellular stress and damage in peripheral tissues. The IL-33 receptor is highly and constitutively expressed on MCs and induces MyD88-TAK1-IKK2-dependent activation of p65/RelA and MAP kinases, which mediate pro-inflammatory cytokine production and amplify MC FcεRI-mediated effector functions, causing allergic reactions. In allergic reactions, heat shock proteins (HSP) are upregulated. The authors demonstrate that HSP90 does not support IL-33-induced activation and MyD88-TAK1-IKK2-dependent activation of p65/RelA and mitogen-activated protein kinases (MAPs). HSP90 acts downstream of these signaling pathways, mediates the stability of the produced cytokine mRNAs and facilitates the resulting cytokine production. IL-33 allows MCs to efficiently produce cytokines via upregulation of HSP90.

Substance P (SP) mediates neurogenic inflammation and pain and contributes to atopic dermatitis in mice through the activation of MCs via G protein-coupled receptor (GPCR)-B2 (MrgprB2, human orthologue MRGPRX2). Some MRGPRX2 agonists activate an additional signaling pathway, involving the recruitment of β-arrestins, which contribute to receptor internalization and desensitization (balanced agonists). In article [4], the authors highlighted that SP causes β-arrestin recruitment and internalization and desensitization of MRGPRX2 in a G protein-independent manner, indicating that SP serves as a balanced agonist for MRGPRX2. This study revealed that MRGPRX2 activation by SP is regulated by β-arrestins and that a highly conserved tyrosine residue within the NPxxY motif of MRGPRX2 contributes to both G protein- and β-mediated arrest. Such findings have important clinical implications, as individuals with this mutation may become resistant to the development of neurogenic inflammation and inflammatory diseases.

Human skin MCs and their mediators participate in the maintenance of tissue homeostasis and regulate the recruitment and activity of immunoregulatory cells involved in the pathogenesis of skin diseases. Review [5] outlines the current knowledge on the bidirectional interactions between human MCs and CD8 T cells and analyzes the alteration in their communication in the context of some skin disorders in which these cells have been found to be altered. Crosstalk between immune cells is important as a potential approach to new therapeutic strategies.

The tumor immune microenvironment is largely orchestrated by immune cells through which tumors acquire resistance against the immune system. Among these cells are MCs and dendritic cells, both of which have enormous capabilities to shape the immune microenvironment. In review [6], the authors discussed how MCs and dendritic cells may modulate solid tumor resistance. Such a modulation could be used in new immunotherapeutic modalities. The authors suggested that the therapeutic power of MCs in cancer treatment may lie in their ability to trigger a much more robust and long-lasting antitumor immunity against solid tumors that have so far resisted immunotherapeutic interventions.

In review [7], the authors provided an overview of the functions of MCs in health and disease. They discussed how mouse models of MC deficiency have become useful tools in establishing MCs as a potential cellular target for the treatment of inflammatory disorders. MCs contribute to maintaining the homeostatic balance between the host and resident microbiota and are involved in the crosstalk between resident and recruited hematopoietic cells.

Review [8] provided a classification of MC disorders, indicated the molecular aspects of MC signaling, elucidated the underlying genetic defects and provided approaches to targeted therapies that may benefit the relevant patients. Some MC disorders are associated with specific genetic mutations. Such disorders include cutaneous mastocytosis, systemic mastocytosis and its variants and clonal MC activation disorders or syndromes. Some disorders are idiopathic in that their molecular pathogenesis and evolution are unclear.

In review [9], the authors discussed the role of extracellular vesicles (EVs) in intercellular communication. MCs are a source of EVs, which transmit biological functions. MC-derived EVs can interact with and influence other cells located in nearby or distant sites, including other types of immune cells, neurons and vascular cells, and can modulate inflammation, allergic response and tumor progression. The authors summarized the most recent data regarding the ability of MCs to release or respond to EVs. EVs derived from MCs located in the tumor microenvironment can enhance tumor cell proliferation and migration, as well as induce angiogenesis, stimulate the antitumor immune response and recruit fibroblasts and macrophages. Tumor-cell-derived EVs can activate MCs and enhance their ability to migrate and release specific cytokines.

Finally, review [10] summarized the current knowledge on the impact of disturbed hemostatic and erythropoietic balance in patients with activated MCs and described the treatment options. MC disease is an epigenetically and genetically determined pathology with potentially very different clinical manifestations in various systems and tissues due to the inappropriate release of mediators as well as the accumulation of morphologically normal or altered mast cells. Disturbances in hemostasis and erythropoiesis often occur in the primary disease of MCs. The most aberrant erythropoiesis in primary MC disease is due to abnormal activation of MCs.

The articles collected in this Special Issue contribute to adding knowledge of the functionality of MCs and heterogeneous and multifaceted cells, shaped by the different microenvironments in which they mature. From this perspective, controlling MC activation or stabilization constitutes a powerful tool in regulating tissue homeostasis and pathogen clearance. MCs are becoming increasingly apparent as potential targets for drugs aimed at a wide range of diseases.
